# The reality of cancer care for many

**DOI:** 10.3332/ecancer.2023.127

**Published:** 2023-07-11

**Authors:** Richard Sullivan, Eduardo Cazap

**Affiliations:** 1 Professor, Cancer & Global Health, Chair ecancer Trustees, Institute of Cancer Policy, King’s College London, London, UK; 2 Editor-in-Chief, ecancer, 13 King Square Avenue, Bristol, BS2 8HU, UK and President, Latin American and Caribbean Society of Medical Oncology (SLACOM), Buenos Aires, Argentina

**Keywords:** cancer medicines, global cancer care, Malawi, disparities, access to care, LMICs

## Abstract

The global literature is heavy with technical documents on how we should deliver cancer care, as well as policy discourses and studies on cancer services and systems. But it is woefully short on the reality of lived experiences. “Adaptations to Deliver Chemotherapy in a District Hospital: Successes and Challenges” from Malawian colleagues based in the Kumuzu University of Health Sciences in the District Hospital of the same name shines a much needed light of realism on the lived experiences of cancer. Why is this letter so important? Because it is much more than a narrative about a district hospital in a low resource country struggling to deliver basic cancer care, it is about the intricate relationship and trade-offs between patients and cancer carers in all resource constrained settings. It bears witness to a reality that feels very far away from the shining bright lights of modern cancer care with all its attendant technological trappings and choices.

“Adaptations to Deliver Chemotherapy in a District Hospital: Successes and Challenges” [1] begins, as all important narratives do, with an idea of hope. Cancer treatments are not therapeutic luxuries, they are essential components of a basic healthcare toolkit; be that surgical, radiotherapy or systemic. Old school chemotherapy remains a critically important tool for alleviating suffering from Kaposi’s sarcoma. In the non-curative setting we often fail to get beyond discussions of access to opiates, yet here is a timely reminder that good quality palliative care needs access to a wide range of therapeutic interventions. Carol Humphry, the author of the letter, also exposes the socio-economic reality of most patients. Daily wage earners one pay day away from destitution.

Humphry lays bare the economic reality not just of the cost of chemotherapy but also the biopsies, blood tests, travel costs, lost wages that accompany such treatment. We tend to gloss over the hard fact that for the majority of the world people pay for their cancer care and this has profound negative consequences. Progressive universalism is a nice phrase but many countries are very far from this when it comes to universal health coverage for cancer care. Nearly a decade ago the ACTION study reported on the economic impact of cancer diagnosis from across South East Asia [2]. Of nearly 10,000 patients studied a year after diagnosis, 29% had died (leaving their families with huge economic losses), 48% experienced devastating poverty-inducing financial catastrophe, and just 23% were alive with no financial catastrophe. Yes, some countries have or are attempting to mitigate the economic consequences of cancer, but they remain the exception not the rule [3].

Another folly held to account is the utility and impact of many of our preconceived ideas around models of care. Humphry’s letter reflects reality-stratified care. A reality that dictates what treatments are available and when, a reality of whether the patient can afford to pay, a reality of whether the patient will return, or whether the costs of toxicity-reducing management are really ‘worth it’. The reason why so many patients across Sub-Saharan Africa still receive vincristine and bleomycin for Kaposi’s Sarcoma is not ignorance [4]. Cancer care for many hospitals is making do with what you have.

The letter also recognizes errors, and their consequences, in the face of fatigue, overwhelming numbers of patients and the need to care for so many different types of cancers by too few healthcare professionals. We discuss with gentle ease quality care in privileged Western settings. For those countries undergoing the cancer transition their demographics and burden mean that cancer care delivery requirements are massive and relentless. Quality cancer care within these ecosystems is a much neglected subject that needs to be tempered with better operational science. The real world shows that access, inequities, efficiency (even with fewer resources) can be improved with a greater focus on the science of quality [5].

It’s hard to move through global cancer without tripping up over an initiative to increase access to cancer medicines, *etc*. Yet we are failing to advocate for the foundations of cancer care for example, basic chemotherapy drugs on the WHO essential medicines list [6]. The letter shows that we are a long way away from even providing basic cancer care in too many settings. Whilst most countries now have some sort of national cancer control plan; few translate into realistic operational plans and quality service delivery. This reflects not just national barriers but a failure of the way the global architecture ‘sees’ cancer as a technical issue that can just be solved with more technology and guidance. If anything, the letter’s final parry speaks to a need for a new idea of social justice in cancer care. One that focuses on the needs of the most vulnerable populations and the recognition by the wider development community that cancer is one of the most fundamental global health challenges of our time.

## Conflicts of Interest

None.

## Funding

RS is supported by GACD Grant Access Cancer Care India GACD-025.
